# The Dynamics and Mechanisms of Interleukin-1α and β Nuclear Import

**DOI:** 10.1111/j.1600-0854.2008.00840.x

**Published:** 2008-11-03

**Authors:** Nadia M Luheshi, Nancy J Rothwell, David Brough

**Affiliations:** Faculty of Life Sciences, University of Manchester, Michael Smith BuildingOxford Road, Manchester M13 9PT, UK

**Keywords:** FRAP, interleukin-1, microglia, nuclear import, Ran

## Abstract

Pro-inflammatory members of the interleukin-1 (IL-1) family of cytokines (IL-1α and β) are important mediators of host defense responses to infection but can also exacerbate the damaging inflammation that contributes to major human diseases. IL-1α and β are produced by cells of the innate immune system, such as macrophages, and act largely after their secretion by binding to the type I IL-1 receptor on responsive cells. There is evidence that IL-1α is also a nuclear protein that can act intracellularly. In this study, we report that both IL-1α and IL-1β produced by microglia (central nervous system macrophages) in response to an inflammatory challenge are distributed between the cytosol and the nucleus. Using IL-1-β-galactosidase and IL-1-green fluorescent protein chimeras (analyzed by fluorescence recovery after photobleaching), we demonstrate that nuclear import of IL-1α is exclusively active, requiring a nuclear localization sequence and Ran, while IL-1β nuclear import is entirely passive. These data provide valuable insights into the dynamic regulation of intracellular cytokine trafficking.

Inflammation is generally a beneficial response of an organism to an injury or an infection. However, dysregulated inflammation can worsen disease progression and outcome. Under both circumstances, the pro-inflammatory cytokines interleukin-1α and β (IL-1α/β) are key drivers of inflammation (1).

IL-1α and β are produced by cells of the innate immune system in response to an inflammatory challenge or stress, as precursor proteins termed pro-IL-1α and pro-IL-1β. Pro-IL-1β requires cleavage by the enzyme caspase-1 to produce an active molecule at the type I IL-1 receptor (IL-1R1). In contrast, pro-IL-1α is active at IL-1R1 2, although it also exists as a mature form after cleavage by calpain 3. Most reported effects of these proteins occur after secretion of the active IL-1 molecule through the ‘classical’ IL-1R1 signaling pathway 4. However, several groups report effects of IL-1α that are dependent on its nuclear localization 5–11. IL-1α can therefore be regarded as a dual function cytokine. In contrast, nothing is known about IL-1β as a nuclear protein.

Nuclear import of proteins requires a nuclear localization sequence (NLS). The NLS is bound by cytosolic importins, which facilitate transport across the nuclear pore complex (12). This process is driven by the gradient of Ran-GTP:Ran-GDP (13). IL-1α contains a NLS in its pro-domain (14), thus suggesting an active import mechanism. However, both IL-1β and IL-1α are small enough (each is less than 50 kD) to passively enter the nucleus (15).

In an injured brain, IL-1 can influence inflammation independently of IL-1R1 (16). Thus, we tested the initial hypothesis that pro-IL-1α localized to the nuclei of microglia after an inflammatory challenge. Surprisingly, not only pro-IL-1α but also pro-IL-1β was detected inside the nucleus. We therefore investigated whether the pro-IL-1β nuclear trafficking was active or passive and established the dynamic nature of pro-IL-1 import mechanisms. Using fluorescence recovery after photobleaching (FRAP) (17), we discovered that pro-IL-1α undergoes NLS and Ran-dependent active transport, while pro-IL-1β transport is passive.

## Results and Discussion

### Both pro-IL-1α and pro-IL-1β are intranuclear cytokines in microglia

In the injured brain, microglia are the major source of IL-1α and β^18^, which are thought to act exclusively post-secretion by IL-1R1. However, endogenous IL-1α is reported to localize to the nucleus of many other cell types 19–22, and the subcellular distribution of IL-1α and β in microglia is unknown. We therefore induced endogenous pro-IL-1α and β expression in BV-2 microglia by stimulation with bacterial endotoxin (lipopolysaccharide, LPS, 1 μg/mL, 6 h). Under these conditions, both IL-1 isoforms were present exclusively as the precursors pro-IL-1α and pro-IL-1β (confirmed by immunoblot; Figure S1). Pro-IL-1 subcellular localization was then characterized by immunocytochemistry.

**Figure 1:**
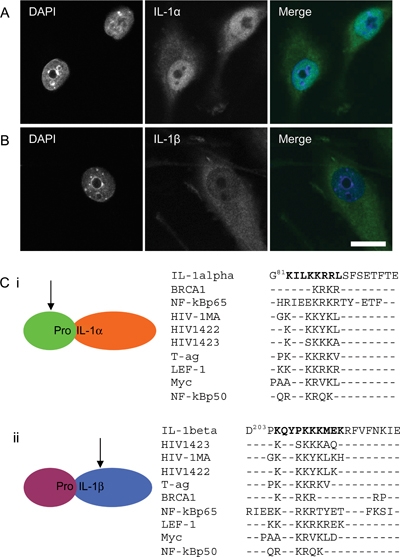
Nuclear localization of pro-IL-1α and β in BV-2 microglia A and B) BV-2 cells (1 × 10^5^ cells/mL) were LPS treated (1 μg/mL, 6 h), immunostained for IL-1α (A) or β (B, green) and co-stained with DAPI (blue). 0.84-μm thick confocal sections of IL-1-immunostained cells were captured at the level of the nucleus. Images are of representative cells from one of three independent cultures. Scale bar represents 20 μm. C) Protein sequence alignments of the NLS in the IL-1α pro-piece (i) and the putative NLS in mature domain of pro-IL-1β (ii, highlighted in bold). BRCA-1, breast cancer 1; HIV-1 MA, HIV-1422 and HIV-1423, HIV viral proteins; LEF-1, lymphoid enhancer-binding factor 1; NF-κBp65, p65 subunit of nuclear factor κB; T-ag, large T antigen from simian virus 40.

Using a confocal microscope to view z-sections at the level of the nucleus, we established that pro-IL-1α and β were detected in both the cytosol and the nuclei of BV-2 microglia after LPS treatment (Figure 1A,B). In agreement with our previous data, we did not detect concentration of endogenous IL-1α or β in any organelle other than the nucleus 23. Nuclear localization of pro-IL-1α and β in BV-2 microglia was confirmed by cell fractionation and immunoblot analysis (Figure S1). We observed identical pro-IL-1α and β nuclear localization in primary microglia (Figure S2). IL-1α and β immunostaining was absent in untreated cells and in microglia isolated from IL-1α/β double knockout mice (Figure S2), confirming the specificity of the intranuclear staining. While endogenously expressed IL-1α is reported widely to localize to cell nuclei, this detection of intranuclear IL-1β was unexpected and raised the question of how IL-1β enters the nucleus.

### NLS-dependent IL-1 trafficking

Previous studies have validated the presence of a NLS in the pro-domain of pro-IL-1α that directs its nuclear localization (14) (Figure 1Ci). However, the mechanisms governing pro-IL-1β nuclear localization remain completely unknown, although it is suggested to contain a putative NLS in the active domain of the protein (24) (Figure 1Cii). We therefore tested whether pro-IL-1β nuclear localization was NLS driven.

The nuclear pore complex is suggested to limit the passive entry of molecules greater than 50 kD (15). Because both pro-IL-1α and β are 31 kD, we generated pro-IL-1-β-galactosidase fusion proteins (147 kD in size) to limit passive entry into the nucleus. COS-7 and HeLa cells were transfected to express β-galactosidase (βgal), βgal with an N-terminal NLS from simian virus 40 large T antigen (nls-βgal) (25), pro-IL-1α-βgal fusion (IL-1α-βgal), pro-IL-1β-βgal (IL-1β-βgal), NLS mutant K85E pro-IL-1α-βgal (mIL-1α-βgal) and a pro-IL-1β-β-galactosidase in which the putative NLS was mutated (K210E, mIL-1β-βgal). Immunoblot of transfected cell lysates confirmed expression of the appropriately sized chimeric proteins (unpublished data).

To quantify the subcellular distribution of the chimeric proteins, wide-field fluorescence images of βgal immunostaining were taken blind and counted. Cells were scored as containing either nuclear, nuclear and cytosolic or cytosolic βgal immunostain (Table 1). These data show that only nls-βgal and IL-1α-βgal had exclusively nuclear localization, while the other chimeric βgal proteins had either a nuclear and cytosolic or exclusively cytosolic distribution (Table 1).

**Table 1:** Quantification of the subcellular localization of IL-1-βgal fusion proteins in COS-7 and HeLa cells^a^

	% nuclear	% nuclear and cytosolic	% cytosolic
COS-7 cells			
βgal	0.00 ± 0.00	66.49 ± 12.58	33.51 ± 12.58
nls-βgal	87.45 ± 4.75***	12.43 ± 4.63*	0.12 ± 0.21
IL-1α-βgal	93.59 ± 3.07***	4.71 ± 3.42**	1.70 ± 1.57
IL-1β-βgal	0.00 ± 0.00	61.01 ± 15.24	38.99 ± 15.24
Mutant IL-1α-βgal	0.00 ± 0.00	34.94 ± 39.34	65.06 ± 39.34
Mutant IL-1β-βgal	0.00 ± 0.00	59.90 ± 18.58	40.10 ± 18.58
HeLa cells			
βgal	0.00 ± 0.00	41.43 ± 7.45	58.57 ± 7.45
nls-βgal	96.69 ± 1.42***	3.02 ± 1.15***	0.29 ± 0.28***
IL-1α-βgal	96.36 ± 1.67***	3.64 ± 1.67***	0.00 ± 0.00***
IL-1β-βgal	0.00 ± 0.00	54.26 ± 8.55	45.74 ± 8.55
Mutant IL-1α-βgal	0.00 ± 0.00	7.24 ± 2.02***	92.76 ± 2.02***
Mutant IL-1β-βgal	0.00 ± 0.00	53.93 ± 13.99	46.07 ± 13.99

a Blind wide-field fluorescence images were captured of the βgal immunostain for at least 50 IL-1-βgal-expressing cells. Individual cells were scored as containing nuclear, nuclear and cytosolic or cytosolic βgal fluorescence. The percentage of IL-1-βgal-expressing cells with each of these three βgal subcellular localizations was then calculated.

*** p < 0.001,

** p < 0.01,

* < 0.05, One-wayanovawith*post hoc*Bonferroni's multiple comparison test, all comparisons are to localization of βgal construct. Data shown are mean ± SD of three experiments.

While the detection of βgal inside COS-7 and HeLa nuclei when expressed alone was surprising, nuclear entry of βgal has been observed previously (26). This could represent βgal that is trapped inside nuclei on reformation of the nuclear membrane after mitosis or the presence of nuclear pore complexes of higher permeability than the often quoted 50 kD limit 27,28242.

**Figure 2:**
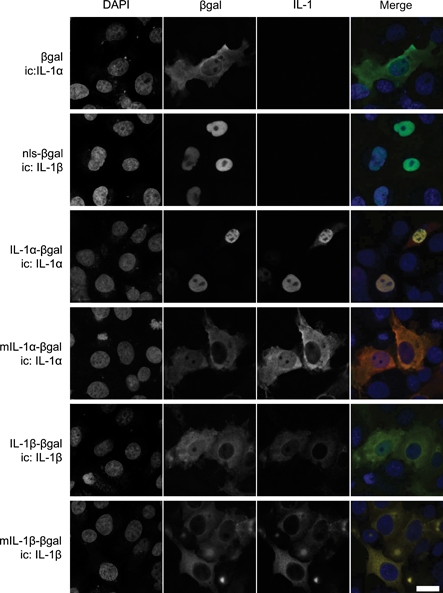
Localization of WT and NLS mutant IL-1-βgal fusion proteins in COS-7 cells Confocal images of IL-1-βgal fusion protein expressing COS-7 cells immunostained for IL-1α or β (red) and βgal (green), co-stained with DAPI (blue). Whether cells were IL-1α or β immunostained is indicated at the left of each panel (ic). 0.84-μm thick confocal sections of immunostained cells were captured at the level of the nucleus. Images are of representative cells from one of three independent cultures. Scale bar represents 20 μm.

Nuclear localization of IL-1α-βgal was abolished by mutation of the NLS, while the subcellular distribution of IL-1β-βgal was not affected by putative NLS mutation and was the same as βgal when expressed alone. Similar results were obtained when fusion proteins were expressed in HeLa cells (Table 1). These effects on nuclear localization are further illustrated by representative confocal micrographs (Figure 2) of transfected COS-7 cells and suggest that the putative NLS of pro-IL-1β is not an NLS and that its nuclear localization may not be regulated by an active process.

### Dynamic nuclear import of IL-1

To further establish the mechanisms of nuclear IL-1 trafficking, we utilized live cell confocal microscopy to study the dynamic nature of IL-1 transport. The wild-type (WT) and mutant forms of pro-IL-1 used above were subcloned into a green fluorescent protein (GFP) expression vector and were used to transiently transfect COS-7 cells. Immunoblot of transfected cell lysates confirmed that the IL-1-GFP chimeras were the correct size (unpublished data).

**Figure 3:**
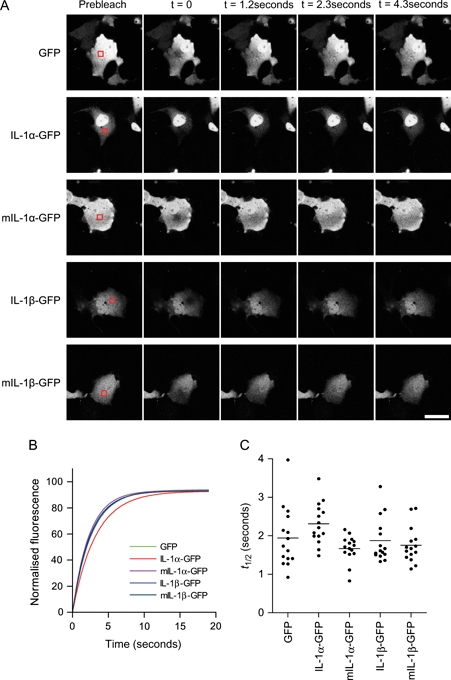
Cytoplasmic FRAP on IL-1-GFP fusion proteins in COS-7 cells A) GFP fusions were overexpressed in COS-7 cells and imaged by live cell confocal microscopy. All images shown are 0.84-μm thick confocal sections through cells at the level of the nucleus. A cytoplasmic ROI was defined (red square) and bleached, and fluorescence recovery was followed in the bleached region. Scale bar represents 40 μm. B) A single-phase exponential association was fit to the fluorescence recovery in the bleach region for each cell by nonlinear regression. Mean recovery curves are shown for all GFP fusion constructs. C) The *t*_1/2_ for fluorescence recovery in individual cells is represented. *n* = 15 cells per construct.

We first characterized cytoplasmic mobility of IL-1-GFP fusion protein by FRAP (Figure 3). A cytoplasmic region of interest (ROI) was defined and bleached, and the kinetics fluorescence recovery was recorded from the bleached region (Figure 3A). The half time (*t*_1/2_) for recovery of cytoplasmic fluorescence was obtained by fitting a one-phase exponential association to the fluorescence recovery curve from the bleached region as described previously (29) (Figure 3B,C). The fraction of IL-1-GFP fusion proteins that were free to diffuse (the mobile fraction, Mf) was as mobile as GFP alone (Figure 3C), indicating that this fraction was freely diffusible in the cytoplasm. However, the IL-1α-GFP Mf was significantly smaller than that of GFP alone (Table 2).

**Table 2:** Cytoplasmic Mfs of IL-1-GFP fusion proteins in COS-7 cells^a^

Construct	Mf (%)
GFP	77.7 ± 7.1
IL-1α-GFP	68.4 ± 11.5*
mIL-1α-GFP	77.9 ± 9.7
IL-1β-GFP	83.0 ± 7.2
mIL-1β-GFP	79.7 ± 4.7

a The cytoplasmic Mfs of IL-1-GFP fusion proteins in COS-7 cells were assessed by FRAP.

* p < 0.05 one-way anovawith *post hoc*Bonferroni's multiple comparison test versus GFP. Data shown are mean ± SD of 15 cells per construct.

**Figure 4:**
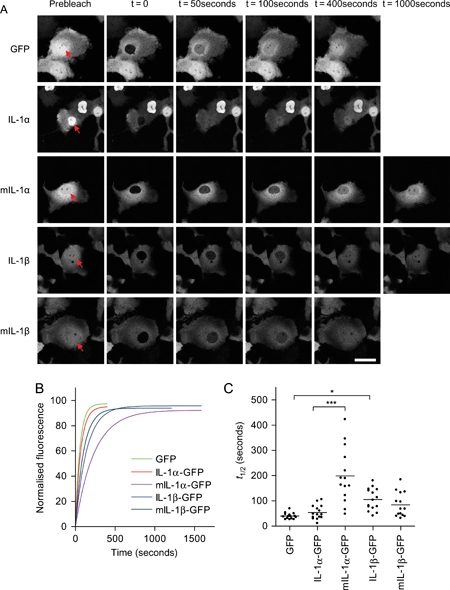
Nuclear FRAP on IL-1-GFP fusion proteins in transfected COS-7 cells A) An intranuclear ROI was defined and bleached, and fluorescence recovery was followed in the bleached nucleus (red arrow). Scale bar represents 40 μm. B) A single-phase exponential association was fit to the fluorescence recovery in the nucleus for each cell by nonlinear regression. Mean nuclear fluorescence recovery curves are shown for all GFP fusion constructs. C) The *t*_1/2_s for fluorescence recovery in individual cells are represented. *n*≥ 14 cells per construct, *p < 0.05, ***p < 0.001, one-way anovawith *post hoc*Bonferroni's multiple comparison test.

We next utilized FRAP to measure IL-1-GFP nuclear import. A ROI that was within the boundary of the COS-7 cell nuclear envelope was selected. Bleaching this nuclear ROI completely depleted nuclear fluorescence. The fluorescence recovery within the nucleus was then recorded and is shown for representative cells for each IL-1-GFP chimera and GFP alone (Figure 4A). The *t*_1/2_ for nuclear fluorescence recovery was again obtained by fitting a one-phase exponential association to the recovery curve. Figure 4B shows the mean exponential recovery curve fit for each IL-1-GFP, and the *t*_1/2_s are plotted in Figure 4C.

IL-1α-GFP nuclear import occurred at a similar rate to that of GFP alone despite being twice the size (IL-1α-GFP *t*_1/2_ = 53.7 ± 27.5 seconds, GFP *t*_1/2_ = 39.7 ± 11.9 seconds). Nuclear import was significantly slowed by mutation of the NLS in pro-IL-1α (mIL-1α-GFP *t*_1/2_ = 198.7 ± 108.8 seconds, p < 0.001 versus IL-1α-GFP), consistent with a reduced efficiency of active nuclear import. Mutation of the IL-1α NLS also led to a redistribution of IL-1α-GFP from the nucleus to the cytoplasm (compare IL-1α-GFP and mIL-1α-GFP localizations in Figure 4A). In contrast, IL-1β-GFP import was significantly slower than that of GFP alone (IL-1β*t*_1/2_ = 105.1 ± 43.3 seconds, p < 0.05) and was unaffected by mutation of the putative NLS (mIL-1β*t*_1/2_ = 84.3 ± 48.4 seconds). This, combined with the IL-1-βgal study above, indicates that IL-1β import occurs independently of this NLS and adds weight to the hypothesis that IL-1β import, unlike IL-1α import, occurs by passive diffusion.

**Figure 5:**
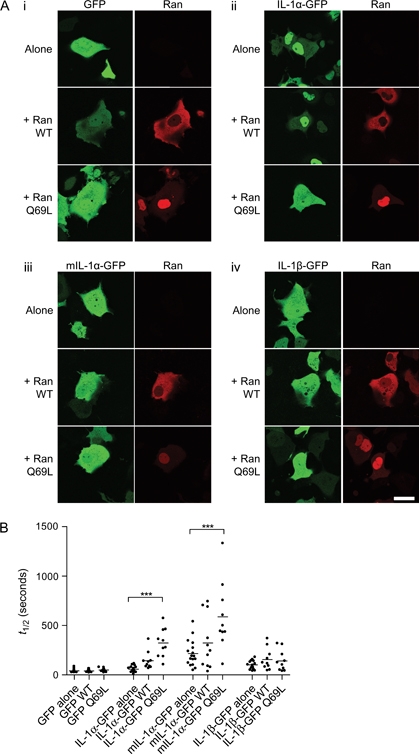
The effect of mCherry-RanWT and -RanQ69L on IL-1-GFP nuclear localization and import A) Confocal slices at the level of the nucleus of COS-7 cells expressing GFP (i) IL-1α-GFP (ii) mIL-1α-GFP (iii) or IL-1β-GFP (iv) constructs alone or with mCherry-RanWT or RanQ69L. GFP (green) and mCherry (red) localizations are shown. Scale bar represents 40 μm. B) *t*_1/2_s for nuclear import of IL-1-GFP fusion proteins following nuclear photobleaching when constructs were expressed alone or with RanWT (WT) or RanQ69L (Q69L). *n*≥ 10 cells per condition, ***p < 0.001, one-way anovawith *post hoc*Bonferroni's multiple comparison test.

To confirm that IL-1α and β utilize different mechanisms for nuclear import, we coexpressed IL-1-GFP fusions with a dominant-negative isoform of Ran, RanQ69L, which lacks the ability to hydrolyse GTP (30). RanQ69L is known to inhibit active nuclear import but should leave import by passive diffusion unaffected (13). To identify Ran-expressing cells, we fused WT and Q69L Ran to mCherry. When expressed, mCherry-RanWT was predominantly cytosolic with some in the nucleus, whereas mCherry-RanQ69L was strongly nuclear. While overexpression of RanWT had no effect on the localization of any IL-1-GFP construct (Figure 5Ai–iv), RanQ69L expression reduced the nuclear localization of IL-1α-GFP (Figure 5Aii).

We then carried out nuclear FRAP on IL-1-GFP-positive cells coexpressing mCherry-Ran. As expected, overexpression of RanWT or RanQ69L had no effect on the passive diffusion of GFP into the nucleus (Figure 5B). In contrast, IL-1α-GFP nuclear import was significantly impaired by the overexpression of RanQ69L (*t*_1/2_ increased from 58.1 ± 29.8 to 323.3 ± 145.1 seconds, p < 0.001 versus IL-1α-GFP expressed alone). Overexpression of RanWT did not significantly inhibit IL-1α-GFP import. mIL-1α-GFP nuclear import was also significantly inhibited by RanQ69L overexpression (*t*_1/2_ increased from 215.1 ± 131.7 seconds to 587.1 ± 340.9 seconds, p < 0.001). This suggests that the slow nuclear import of mIL-1α-GFP observed in Figure 3C represents a reduction in the efficiency of active import but that IL-1α does not enter the nucleus passively.

IL-1β-GFP import was unaffected by overexpression of RanQ69L (Figure 5B), confirming that IL-1α and β do indeed use different mechanisms for nuclear import – active, NLS-driven and Ran-dependent active import for IL-1α and passive import for IL-1β.

### IL-1β: an intranuclear cytokine?

We have shown in this study for the first time that both IL-1α and IL-1β are intranuclear cytokines in microglia. Because IL-1β nuclear localization had not previously been reported and the mechanism of IL-1β import remained unknown, we investigated whether IL-1α and β utilized similar nuclear import mechanisms. We have found that this is not the case. IL-1α can drive βgal nuclear import in an NLS-dependent fashion (Table 1 and Figure 2) and has an import rate that is dependent on the integrity of its NLS and upon an unperturbed nucleocytoplasmic Ran cycle (Figures 4 and 5). This fits well with previous reports that IL-1α nuclear import is NLS dependent 6,9,14 and confirms that IL-1α import uses the classical nuclear import machinery. The extremely slow rate of NLS mutant IL-1α import in comparison to the similarly sized IL-1β, and the further slowing of this rate by RanQ69L overexpression, suggests that IL-1α only uses this active import mechanism and perhaps that the engagement by proteins of the active import machinery can inhibit their passive diffusion into the nucleus. The reduced IL-1α cytosolic Mf (Table 2) could be accounted for by this interaction with the import machinery.

In contrast, IL-1β failed to drive βgal nuclear import, and mutation of the putative NLS in its mature domain had no effect either on the localization of IL-1β-βgal or on the import rate of IL-1β-GFP (Table 1 and Figures 2 and 4). This suggested that the IL-1β putative NLS was not in fact capable of engaging with nuclear import machinery. RanQ69L overexpression had no effect on the import of IL-1β, confirming that IL-1β nuclear import occurs passively, in contrast to IL-1α active import (Figure 5).

IL-1α-GFP and IL-1-β-GFP import rates were remarkably similar (Figure 4), given that the two proteins enter the nucleus by different mechanisms. It appears that the active import of IL-1α serves to concentrate IL-1α inside the nucleus, against a concentration gradient, rather than to speed up its import.

Recent reports that the IL-1 family members IL-33 and IL-1F7b have intranuclear actions in IL-1-expressing cells 31,32 support the hypothesis that many IL-1 family members, and not just IL-1α, may be dual function cytokines. Our finding that IL-1β, like IL-1α, is an intranuclear cytokine raises questions as to whether IL-1β also has intranuclear actions. Although IL-1α and β mature domains share structural similarity and both bind to and activate IL-1R1 (1), the IL-1α pro-domain is responsible for many of the reported IL-1α intranuclear actions 6–10,33, and this domain shares little sequence homology with the pro-domain of IL-1β. Further investigation is now required to determine whether IL-1β has a separate set of intranuclear actions.

## Materials and Methods

### Production of IL-1-βgal, IL-1-GFP and mCherry-Ran fusion proteins

Murine pro-IL-1α and β without STOP codons were polymerase chain reaction (PCR) amplified from image clones BC003727 and BC011437 (MRC Gene Service) and inserted into pL28. K85E pro-IL-1α and K210E pro-IL-1β were generated using the Quickchange® II site-directed mutagenesis kit (Stratagene). WT and mutant pro-IL-1α and β were then subcloned into pEGFP-N1. βgal was subcloned from pL38-TaulacZ into pL28, and WT and mutant IL-1α and β were fused to the 5′ end of βgal prior to subcloning of the IL-1-βgal fusions into pVITRO2-neo-mcs. βgal and nls-βgal were subcloned from pSLX-lacZ2 and pWhere, respectively, into pVITRO1-neo-mcs. WT and Q69L human Ran-GTPase were PCR amplified from pJG4-5-Ran and inserted into pcDNA3.1(-)-mCherry. The presence of unmutated inserts in recombinant plasmids was confirmed by sequencing.

### Cell culture

BV-2, COS-7 and HeLa cells were maintained in DMEM (Cambrex Biosciences) with antibiotics (100 μg/mL streptomycin and 100 IU penicillin) plus 2.5% FBS (PAA laboratories), 10% FBS or 10% FBS with nonessential amino acids, respectively. LPS treatment (1 μg/mL, 6 h, *Escherichia coli*026:B6) was used to induce IL-1 synthesis in BV-2 cells (1 × 10^5^ cells/mL). COS-7 and HeLa cells were transfected with IL-1-GFP, IL-1-βgal and mCherry-Ran fusions with lipofectamine 2000 (Invitrogen).

### Immunocytochemistry

Cells on coverslips were fixed with 4% paraformaldehyde/4% sucrose, permeabilized with 0.1% Triton-X-100 and then quenched with 0.25% NH_4_Cl. A blocking step with 5% BSA/5% normal donkey serum (Stratech Scientific; ‘block solution’) was used prior to the incubation of cells with goat anti-mouse IL-1α or β antibody, 1 μg/mL (R&D Systems), or fluorescein isothiocyanate-conjugated rabbit anti-βgal, 20 μg/mL (Abcam) in block solution. Alexa Fluor 488 or 594 donkey anti-goat (1 μg/mL; Invitrogen) in block solution was used for primary antibody detection. The coverslips were mounted in ProLong Gold with 4′,6-diamidino-2-phenylindole, (DAPI, Invitrogen).

### Microscopy

All microscopy was carried out at the Core Bioimaging Facility, Faculty of Life Sciences, University of Manchester.

Confocal microscopy was carried out on a Leica SP5 AOBS tandem head confocal using a blue diode, argon laser (20% laser power utilized for imaging fixed cells) and orange He/Ne laser, a ×63/1.40 HCX PL Apo objective and a pinhole of 1 airy unit. Leica LAS AFsoftware was utilized for image acquisition and FRAP analysis.

Wide-field images were captured using an Olympus BX51 upright wide-field microscope with a ×40/1.00 UPlan Apo objective and a Coolsnap ES camera (Photometrics) through MetaVuesoftware (Molecular Devices). All wide-field images were captured using the same exposure and image scaling settings, and image scaling was adjusted to exclude background immunostaining. Offline image analysis used ImageJsoftware (http://rsb.info.gov/ij/).

### Fluorescence recovery after photobleaching

FRAP experiments were carried out 18–36 h after COS-7 cell transfection. Cells were maintained at 37°C with 5% CO_2_ throughout experiments. Confocal settings were as follows; argon laser power was set to 80%, scan speed was 700 Hz unidirectional, zoom was ×1.7 and GFP fluorescence was imaged during the experiment utilizing 6–8% of the 488 laser line power.

For cytoplasmic FRAP, a cytoplasmic ROI (9.6 × 9.1 μm) was defined. Twenty prebleach 256 × 256 pixel images were acquired followed by 10 ROI bleach cycles over 3.8 seconds using 100% of the 458, 476 and 488 laser lines. Recovery was followed over a further 50 post-bleach frames, all acquired at maximum speed (2.6 frames/second).

For nuclear FRAP, 20 pre-bleach 512 × 512 pixel images were acquired at maximum speed (1.3 frames/second) prior to 15 ROI photobleach cycles of an intranuclear ROI over 11.5 seconds as above. Because of rapid intranuclear IL-1-GFP diffusion, this led to complete nuclear bleach. Recovery was then followed by taking images every 2–4 seconds until the intranuclear fluorescence asymptote was reached.

Mobility (*t*_1/2_) and Mfs were calculated as described above and reported previously 29,34.

### Data analysis

All statistical analyses used GraphPad Prismversion 4.00 for Windows from GraphPad Software. Differences between groups were identified using one-way analysis of variance (ANOVA) with *post hoc*Bonferroni's multiple comparison test. All data are expressed as mean ± SD of at least three independent experiments.

### Online supporting information

Figure S1 shows immunoblot analysis and lactate dehydrogenase assay of LPS-treated BV-2 cell cytoplasmic and nuclear fractions. Figure S2 shows IL-1 immunostaining of untreated and LPS-treated primary WT and IL-1α/β-deficient microglia.

## Supporting Information

**Figure S1: Detection of pro-IL-1α and β in BV-2 microglial nuclear fractions.** IL-1α and β immunoblot (A), poly (ADP-ribose) polymerase (PARP, nuclear marker protein) immunoblot (B) and lactate dehydrogenase (LDH, cytosolic marker protein) assay (C) of nuclear and cytoplasmic fractions from BV-2 cells treated with LPS (1 μg/mL, 1–24 h). All data shown are from one representative experiment of three independent experiments. Cells were lysed (10 min, 4°C) in 1% Triton-X-100 (v/v) with 50 mM Tris–Hcl, pH 7.5, 50 mM β glycerophosphate, 1 mM sodium orthovanadate, 5 mM sodium fluoride and protease inhibitors. Nuclei were isolated by centrifugation (5 min, 1500 × g, 4°C). Remaining debris was removed from supernatants by a further centrifugation (10 min, 10 000 × g, 4°C). Supernatants were then saved as cytoplasmic fractions. Nuclear pellets were washed twice prior to the extraction of nuclear proteins into lysis buffer with 0.5 M NaCl. Remaining nuclear debris was pelleted by centrifugation (10 min, 10 000 × g, 4°C), and the supernatant was saved as the nuclear fraction.

**Figure S2: IL-1 expression and localization in primary microglia.** A and B) Wide-field images of primary microglia from WT (C57, A) and IL-1α/β-deficient (IL-1α/β KO, B) microglia, cultured as described previously (35), treated with LPS (1 μg/mL, 6 h), immunostained for IL-1α or β and costained with DAPI. Images are of representative cells from one of two independent cultures. Scale bar represents 40 μm.
