# Supplementary figures and images for: The Dynamics and Mechanisms of Interleukin-1α and β Nuclear Import
Source: Traffic. 2008 Nov 3;10(1):16–25. doi: 10.1111/j.1600-0854.2008.00840.x (PMC2682621; doi:10.1111/j.1600-0854.2008.00840.x)

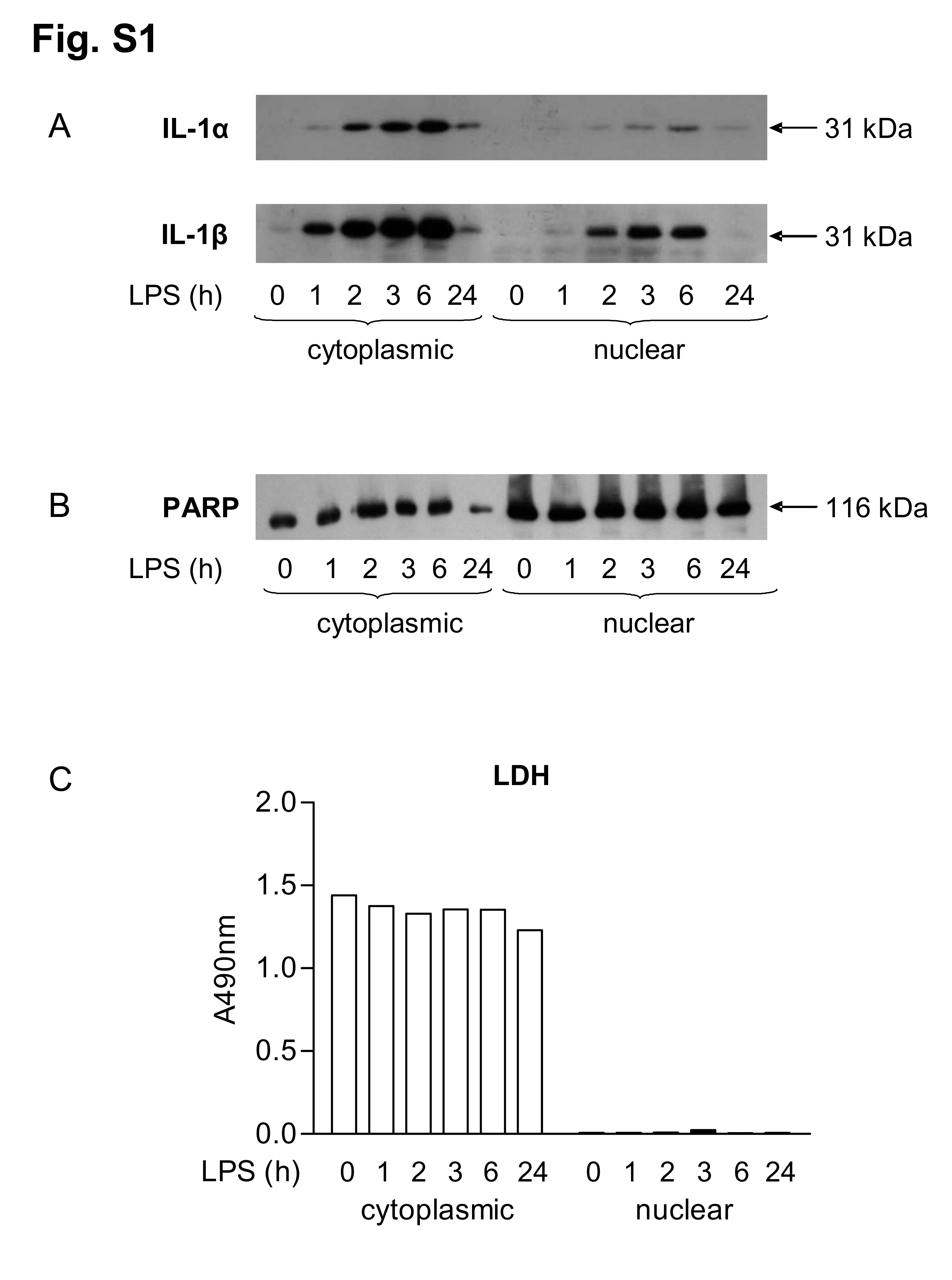

Supplement: Supplementary file 1 [file tra0010-0016-SD1.tif]

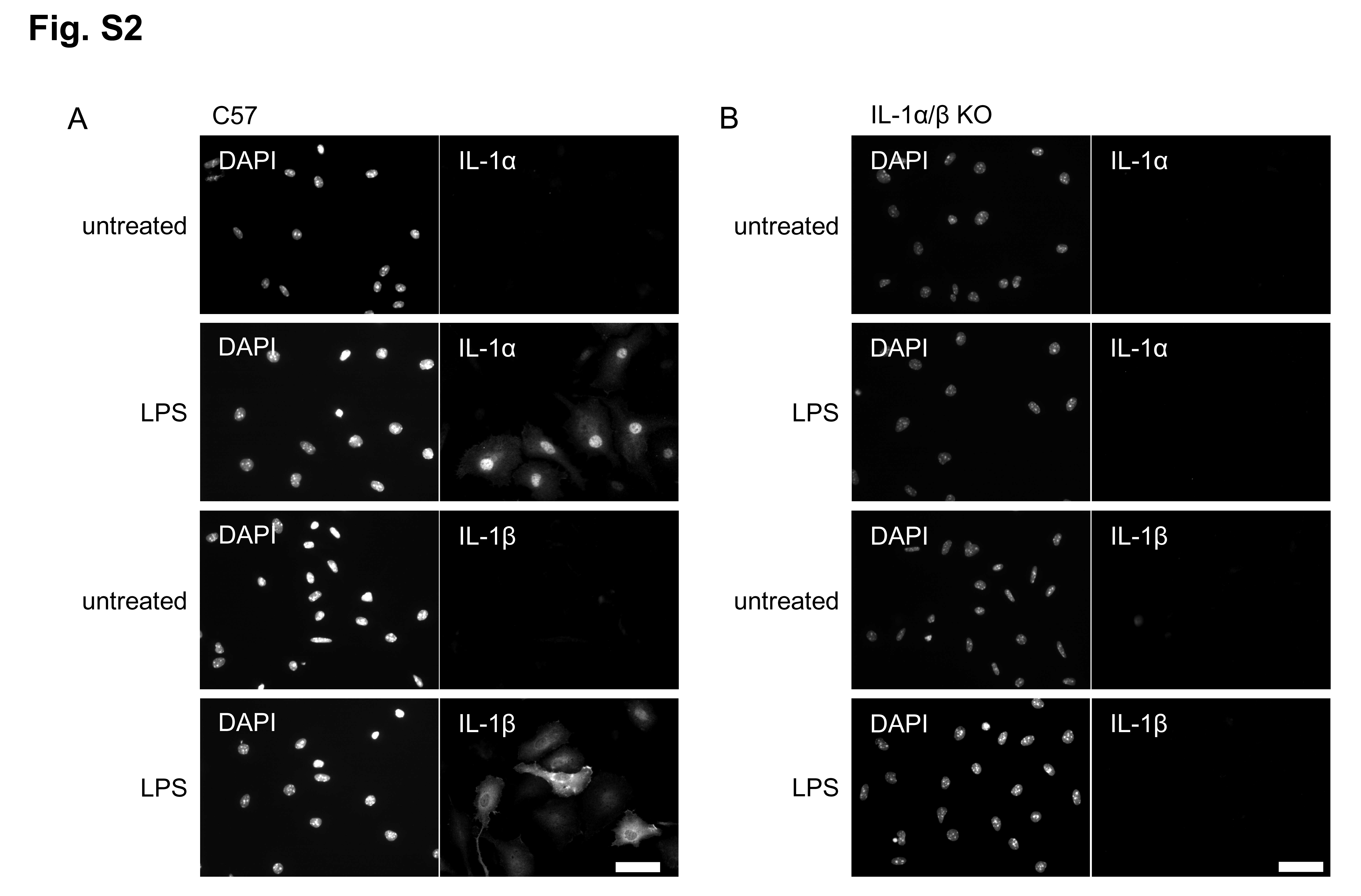

Supplement: Supplementary file 2 [file tra0010-0016-SD2.tif]
